# *Dendropanax Morbifera* Extract-Mediated ZnO Nanoparticles Loaded with Indole-3-Carbinol for Enhancement of Anticancer Efficacy in the A549 Human Lung Carcinoma Cell Line

**DOI:** 10.3390/ma13143197

**Published:** 2020-07-17

**Authors:** Esrat Jahan Rupa, Lakshminarayanan Arunkumar, Yaxi Han, Jong Pyo Kang, Jong Chan Ahn, Seok-Kyu Jung, Mia Kim, Jong Yun Kim, Deok-Chun Yang, Gyong Jai Lee

**Affiliations:** 1 Department of Oriental Medicinal Biotechnology, College of Life Science, Kyung Hee University, Yongin-si 17104, Gyeonggi-do, Korea; eshratrupa91@gmail.com (E.J.R.); arunmsc80@khu.ac.kr (L.A.); navycki@gmail.com (Y.H.); seokkyujung@gmail.com (S.-K.J.); 2 Department of Cardiovascular and Neurologic Diseases, College of Korean Medicine, Kyung Hee University, Seoul 100011, Korea; kangjongpyo@naver.com (J.P.K.); jongchanahn@gmail.com (J.C.A.); hyuntemia@hanmail.net (M.K.); 3 Saerom Hanbang R&D Center, 76, Cheonseok-gil, Geumcheon-myeon, Naju-si 520010, Jeollanam-do, Korea; sr8919@naver.com; 4 SD Leo R&D Center, 9-16, Yeonmujang 5-gil, Seongdong-gu, Seoul 100011, Korea

**Keywords:** *dendropanax morbifera*, zinc nanoparticles, nanoemulsion, indole-3-carbinol, anti-cancer activity, cytotoxicity, lung cancer

## Abstract

*Dendropanax morbifera* is a versatile plant that has been used as a herbal medicine due to its various useful medicinal effects. To protect its active component from biological stress and increase its drug efficacy as well as drug bioavailability, nanoemulsion was prepared. *Dendropanax morbifera* zinc oxide nanoparticles (*DM*-ZnO NPs) were synthesized using the plant extract via the co-precipitation method and loaded with active indole-3-carbinol for nanoemulsion formulation using the ultrasonication process. Field emission transmission electron microscope revealed the flower shape of the *Dendropanax morbifera* indole-3-carbinol zinc oxide nanoemulsion (*DM*-ZnO-I3C-NE). In contrast, *DM*-ZnO NPs showed a spheroid shape that coincides agreeably with field emission electron scanning microscope. The hydrodynamic sizes by dynamic light scattering are about 65 ± 3 nm and 239.6 ± 6 nm and the crystallite sizes from X-ray diffraction are 11.52 nm and 16.07 nm for *DM*-ZnO NPs and *DM*-ZnO-I3C-NE, respectively. In vitro analysis revealed the cytotoxicity of *DM*-ZnO-I3C-NE against a human lung cancer cell line (A549) at 12.5 µg/mL as well as reactive oxygen species (ROS) production. The *DM*-ZnO-I3C-NE-induced ROS generation level was higher than that of *DM*-ZnO NPs and free indole-3-carbinol. The synergistic effect of *DM*-ZnO and indole-3-carbinol indicates *DM*-ZnO-I3C-NE as a potential candidate for future lung cancer drug and could be scope for functional food.

## 1. Introduction

Cancer shows the highest mortality rate worldwide, which is due in part to mutations that enable cancer cells to survive and spread to other organs [1]. Approximately 40% of patients with lung cancer worldwide die within one year of diagnosis [2]. Nano-based drug delivery systems (DDS) have shown great promise due to advantages including the higher bioavailability, targeted specificity, and uptake mechanisms of absorptive endocytosis. The integration of green phytoextracts and nano-biotechnology approaches has contributed to improvements in DDS [3]. Plant extracts have been considered safe, cheap, and free from hazards associated with chemical synthesis. Recently, several metal nanoparticles using gold, silver, and zinc were synthesized using phytoextracts and showed efficacy as significant pharmacological active compounds [4]. Plant extracts provide a platform for metal nanoparticles and thereby increase their efficiency in the target-specific environment; furthermore, the plant extract acts as a natural capping and carrier for active ingredients and metal particles [5]. Among the various metal nanoparticles, the zinc oxide nanoparticle has been commonly applied in the field of drug delivery due to its unique property of being active in intracellular and extracellular routes [6]. From prehistoric times, medicinal plants have found their uniqueness in traditional medicine practices for various diseases. The integration of green chemistry has provided an ample platform for expanding the modern carrier system using these traditional medicine plants [7].

In our recent approach, we used the traditional *Dendropanax morbifera* (*DM*) for synthesizing zinc oxide nanoparticles and further entrapped with them with indole-3-carbinol (I3C) using the oil-in-water (O/W) nanoemulsion technique. *Dendropanax* is a genus of flowering plants in the family Araliaceae that consists of almost 92 species of evergreen trees and shrubs found at the seashore and islands in the southern part of South Korea. Dendro means “tree” and Panax means “Panacea,” and *DM* falls under the subtropical evergreen panacea family. The leaf, stem, and root of this oriental medicinal plant provides significant remedy for infectious diseases, skin, migraine, and various anti-inflammatory activities [8]. A recent study reported that polyacetylene compounds from *DM* show anti-complementary activity [9]. Based on the multiple properties of *DM*, it has been applied as functional food materials with anti-diabetic, anti-cancer, and anti-atherogenic activities. *DM* extract contains triterpenoids, polyacetylene, and phenolic substances that have been shown to exhibit anti-inflammatory and anti-cancer properties. The information and composition of the extracts still not much explored. However, the biological applications of these substances are under investigation. These plant extracts with nanoformulation have become more prevalent in the past decades, due to the valuable components and nutraceutical properties. Oleifolioside B, a component in *DM,* exhibits anti-cancer activity by activating autophagy and inducing apoptosis through caspase activation [10].

Nano ZnO has gained recent attention in food additives as a GRAS (generally recognized as safe) substance by the US Food and Drug Administration (FDA) [11]. Nano ZnO is comparatively less toxic and inexpensive, with biological functions such as anti-cancer, anti-inflammatory, antibacterial, anti-diabetes, wound healing, and bioimaging activities [12]. Treatment with dissolved ZnO NPs leads to caspase activation and an increase in ROS concentration in intracellular conditions, resulting in the loss of protein and cell apoptosis. Sharma et al. found that dissolved zinc ions play critical roles in the apoptosis signaling pathway. Several studies have investigated the numerous mechanisms and site-specific applications of ZnO nanoparticles (NPs) in various cancer cells [13].

Considering the advantages of *DM* and Zn, synthesizing *DM*-ZnO NPs and nanoemulsion (NE) has been explored for food supplements and cancer treatment. To increase the efficacy of *DM*-ZnO NPs, I3C, another green source and anti-cancer substance, was loaded using non-ionic surfactant and olive oil in an O/W nano emulsification system. We prepared the oil-in-water nanoemulsion by an ultrasonication method for enhancing the solubility of the active component inside *DM* and I3C by trapping the hydrophobic part inside of the hydrophilic part by creating ultrasonic cavitation. Moreover, the lipophilic part solubility will improve in the water form of nanoemulsion that sequentially increases the bioavailability and medicinal properties of the drug.

I3C is found in cruciferous vegetables such as broccoli, cabbages, and cauliflower and shows excellent tumor-blocking initiation capabilities [14]. I3C also serves a novel platform for various chemo-sensitizing applications. Since the half-life of I3C is not of the highest order, it is essential to deliver it at the right site with the active component of the plant to enhance its efficacy as well as increase the solubility using lyophilic components of the plant. In this article, we developed a novel platform for carrying I3C with zinc metal nanoparticle synthesized using high actives involving DM extracts. This study provides an abundant composition of making a novel anti-cancer drug with an additional application as a safe food supplement.

In this article, we carefully synthesized biologically active nanoemulsion and performed structural identification. Morphology was determined using dynamic light scattering (DLS), Fourier transforms infrared analysis (FTIR), field emission transmission electron microscopy (FE-TEM), ultraviolet-visible (UV-Vis) spectrophotometry, and FE-SEM, which aided in the elucidation of the morphological and chemical natures of the NE. *DM*-ZnO NPs were successfully synthesized from *DM* extract, and I3C was entrapped using the oil-in-water nanoemulsion system in which ZnO NPs water solution was used as the aqueous phase and olive oil was used as the oil phase with Tween 80 as a surfactant. The effect of the synthesized nanoformulation was investigated using the A549 lung cancer cell line and the HaCaT human keratinocyte cell line. Furthermore, the anti-cancer activity was checked via measurement of ROS level.

## 2. Materials and Methods

### 2.1. Plant Materials

The *Dendropanax morbifera* sample was collected from of Wando-gun city of South Korea from a 100-year-old tree in the September 2019. A total of 10 kg of plant were collected and extracted.

### 2.2. Chemicals

Zinc nitrate hexahydrate (>98.0%), indole-3-Carbinol (Sigma Aldrich, St. Louis, MO, USA), sodium hydroxide (>98.0%). All media was supplied from Difco, MB Cell (Gangnam-gu, Seoul, Korea). Absolute alcohol was supplied by Samchun Pure Chemical Co. Ltd. (Gyeonggi-do, Korea). Olive oil and Tween 80 were obtained from Samchun Pure Chemical Co. Ltd. The human keratinocyte cell line (HaCaT) and lung cancer cell line (A549) used in this study were obtained from the Korean cell line bank (Seoul, South Korea). RPMI 1640 (Roswell Park Memorial Institute Medium) culture medium was purchased from GenDEPOT Inc. (Barker, TX, USA). Dulbecco’s modified eagle’s medium (DMEM) (Gibson-BRL, Grand Island, NY, USA) with 10% fetal bovine serum (FBS) and 1% penicillin/streptomycin (p/s) (WElGENE Inc., Daegu, Korea) was also used for experiments.

### 2.3. Preparation of DM Extract

The sample was washed to remove any debris and ground into fine particles. Plant powder (10 g) was placed in conical flasks with 100 mL of DW (Distilled water) and autoclaved for 60 min at 100 °C under high pressure to obtain an aqueous extract. The autoclaved extract was filtered with filter paper (Whatman-No.1) and centrifuged at 5000 rpm for 15 min to remove debris. The collected supernatant was kept at 4 °C until experiments.

### 2.4. Synthesis of DM-ZnO NPs from the Extract

The *DM*-ZnO NPs were prepared using the co-precipitation method with minor modifications [15]. Zinc nitrate hexahydrate worked as an oxidizing salt and sodium hydroxide worked as a precipitating precursor. The method involves precipitating nanoparticles, and the purification includes the washout of unreacted zinc nitrate salt and phytochemicals. Initially, 20 mL of 10% *DM* extract (w/v) was mixed with 80 mL of distilled water under stirring, and 0.1 mM (10 mL) of zinc nitrate salt was added to the light brown solution with continuous stirring (500 rpm). Then, the solution was heated up to 65 to 70 °C. Then, 0.2 M (15 mL) aqueous solution of NaOH was added dropwise into the hot solution with continuous stirring over 120 min. During this process, the precipitate begins to accumulate, thicken, and form a uniform mixture. The mixture was allowed to cool down without stirring, after which it was centrifuged at 8000 rpm at 15 min to remove the unreacted substances. A cold-water wash (5 °C) was performed. Solid particles were kept at 60 °C for 4–5 h in the oven for the complete conversion of Zn(OH)_2_ to ZnO NPs as a white *DM*-ZnO-NP powder.

### 2.5. Synthesis of DM-ZnO-I3C NE

**Figure 1:**
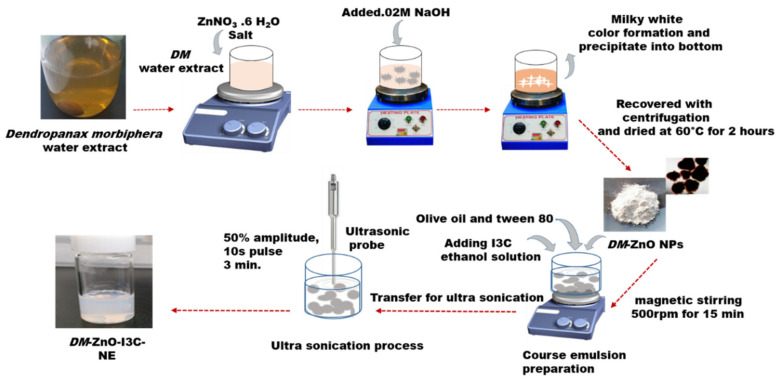
Preparation of *DM*-ZnO-I3C oil in water nanoemulsion using the ultrasonication method.

In this approach, we adapted a higher energy ultrasonication technique for the development of O/W nanoemulsion using the ultrasonication method (Figure 1). This technique involves an amplitude of 50% for 3 min with a pulse rate of 10 sec. I3C was dissolved in 20 μL of ethanol with a mixture of olive oil and Tween 80 as a surfactant. The sonication conditions were selected based on the quantity. The nanoformulation was performed under ice-cold conditions to avoid heat generation due to the cavitation phenomenon. The size improves stability for long storage and helps in the drug-carrying ability of the formulation. Three formulations were prepared using different conditions to check the stability: S1, S2, and S3. All three samples were reviewed, and the stable formulated product was further characterized and underwent efficiency testing.

### 2.6. Characterization

The synthesized nanoemulsion and nanoparticles were characterized via various analytical instruments for measuring size, morphology, and stability.

The green synthesized NP solutions were observed between 200 and 700 nm with a UV-Vis spectrophotometer (Ultrospec TM-2100 pro) for the confirmation of the *DM-ZnO NPs* and *DM*-ZnO-I3C NE.

The surface capping materials of the NPs and NE were determined by FTIR analysis (PerkinElmer Inc., Waltham, MA, USA) between 4000 and 450 cm^−1^. The spectral studies were plotted as transmittance (%) versus wavenumber (cm^−1^).

XRD was analyzed using D8 Advance (Bruker, Karlsruhe, Germany) that confirmed the crystallinity and size of *DM*-ZnO NPs and *DM*-ZnO-I3C-NE in a 2θ range of 20°–80° angle with an operating voltage of 40 kV as well current of 40 mA &Cu-Kα radiation of 1.54 Å.

DLS analysis data were obtained at 25 °C with a zeta potential and particle size using the zeta analyzer and ELSZ-2000 series (Otsuka Electronics Photal, Osaka, Japan), which was used to define the size distribution.

The morphology of the purified metallic NPs and nanoemulsion was examined using a multifunctional 200 kV-operated JEM-2100 F (JEOL, Akishima, Tokyo, Japan). The samples were prepared using a copper grid on which a small amount of the samples was dropped.

In this study, we used an FE-SEM to obtain topographical and elemental information (LEO SUPRA 55, GENESIS 2000 (Carl Zeiss, EDAX); gun: thermal field emission type; resolution: 1.0 nm @15 kV 1.7 nm @1 kV 4.0 nm @0.1 kV; magnification: 12× to 900,000×). FE-SEM provides more transparent and higher regulation than typical SEM.

#### Cell culture

A549 cells were cultured in RPMI 1640 with 10% FBS, 1% penicillin. HaCaT cells were cultured in DMEM with 10% FBS and 1% p/s. Both cell lines were cultured at 37 °C in a humidified incubator with 5% CO_2_ atmosphere.

Cells were seeded in X-well plates at 1 × 10^4^ cell/well. Cells were treated as indicated with various concentrations (0, 3.125, 6.25, 12.5, 25 µg/mL) and incubated for 24 h. Then, cells were treated with 20 µL of 3-(4, 5-dimethyl-2-thiazolyl)-2, 5-diphenyl tetrazolium bromide solution (MTT; 5 mg/mL, PBS; Life Technologies, Eugene, OR, USA) for 4 h at 37 °C. Viable cells convert the MTT solution to purple-colored formazan; the insoluble formazan was dissolved by 100 µL DMSO in each well. Readings were taken at 570 nm with an Enzyme-Linked Immunosorbent Assay (ELISA) reader (Bio-Tek, Instruments, Inc., Winooski, VT, USA).

ROS levels were determined using 2′,7′-dichlorofluorescein (DCF-DA). A549 cells were seeded at 1.0–1.2 × 10^4^ cells per well in X-well plates overnight. Cells were treated with various concentrations of DM-ZnO-I3C-NE for 24 h. Next, 25 μM of the DCFH-DA (2′-7′dichlorofluorescin diacetate) solution was added into each well and samples were incubated for 30 min. The supernatant was discarded, and cells were washed twice with 1 × dPBS (Dulbecco’s phosphate-buffered saline). A multi-model plate reader was used for measurement of the fluorescence intensity by an excitation wavelength of 485 nm and an emission wavelength of 520 nm. ROS generation assays were also performed using *DM*-ZnO-I3C-NE with *DM*-ZnO NPs and I3C at the same concentrations.

## 3. Results and Discussion

### 3.1. Physicochemical Properties of the Synthesized Nano Formulation

To ensure the stability of the nanoemulsion, we synthesized three different concentrations: S1, S2, and S3. All three contained different compositions of the oil and water ratio, as shown in Table 1. The composition of surfactant, water, and oil percentage in each phase mostly determines the O/W (oil in water) or W/O (water in oil) system of nanoformulation. It was recently reported that 50–90% of the aqueous phase and the surfactant concentration should be 1.5–10% in oil in water [16,17]. High percentages of surfactant may cause skin and eye irritation, so we carefully considered 2–10% surfactant concentration. Samples with different proportions of water, oil, and surfactant were tested by checking stability using physical visualization over seven days. Over seven days of observation, the sample showed little creaming for S1 and precipitation for S3. The stability of the particle depends on the non-formation of creaming and precipitation or any phase separation during observation [18]. Due to the misappropriate composition of surfactant, oil, and water, the binding nature of the materials differed. Hence, our formulations of S1 and S3 were not stable for seven days, and so we did not pursue experiments with these samples. Sample S2 showed excellent stability during observation, so we performed another evaluation of its stability.

Sample S2 was centrifuged at 3000 rpm for 30 min, and we did not observe any precipitation during the observation time. To evaluate temperature stability, the formulation was tested at different temperatures (5 °C, 25 °C, 40 °C) for 15 days of observation. S2 showed stability as shown by observing the zeta potential and PDI (Poly dispersity index) value. The low polydispersity value of NE (less than 0.2) indicated efficient formulation of the system as well homogeneity, which may help attain excellent stability [19]. Based on the preliminary results, S2 was chosen for further experiments. *DM*-ZnO-I3C-NE contains 80% aqueous part containing 37.5 mg of *DM*-ZnO NPs and olive oil (8%) as well as a surfactant (7%) and was loaded with 5% I3C (12.5 mg).

### 3.2. UV-Vis Analysis

**Figure 2:**
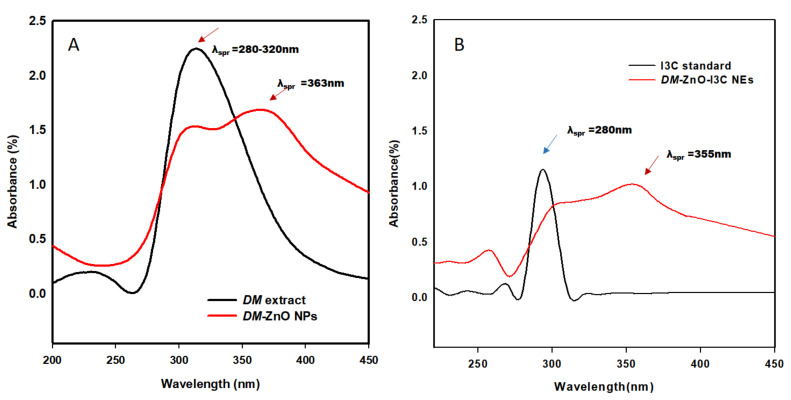
UV-Visible spectra of (**A**) *Dendropanax morbifera* zinc oxide nanoparticles (*DM*-ZnO NPs) with *DM* extract, (**B**) *DM*-ZnO-I3C-NE with standard I3C.

Figure 2 shows the UV absorbance spectra of the plant extract, extract-capped ZnO NPs, I3C-loaded *DM*-ZnO nanoemulsion, and I3C alone. Results indicated that *DM* successfully synthesized ZnO NPs using the co-precipitation method. *DM*-ZnO NPs showed a sharp surface at plasmon resonance wavelength (λ_spr_) at approximately 363 nm, demonstrating the formation of ZnO NPs. The *DM* extract showed a sharp absorption peak around 280–320 nm due to the presence of polyphenol [20,21]. Anthocyanins are highly reactive phenolic compounds. In this case, the anthocyanin molecule may show a characteristic broad band around 312 nm. Therefore, *DM* extract functionalized with ZnO NPs and ZnO-I3C-NE both showed the absorption spectra at 250–380 nm [22,23], which confirmed the presence of *DM* extract in the formulation (Figure 2A). I3C showed a sharp absorbance peak at around 280 nm, and *DM*-ZnO-I3C-NE also showed absorbance at 280 nm, demonstrating the entrapment of I3C during nanoemulsion preparation (Figure 2B). These results were further evidenced by the morphological changes observed by SEM and TEM characterizations and corresponding EDS (Energy dispersive spectroscopy) elemental mapping.

### 3.3. FTIR Spectroscopic Analysis

**Figure 3:**
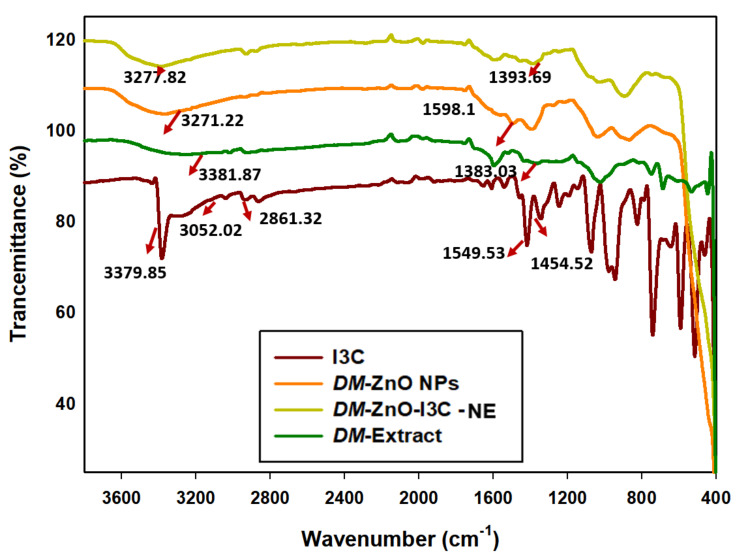
Fourier transforms infrared analysis (FTIR) for I3C standard (Red), *DM*-ZnO NPs (orange) *DM*-ZnO-I3C-NE (yellow) and *DM* extract (green).

FTIR spectra of *DM* plant, I3C, *DM*-ZnO NPs, and *DM*-ZnO-I3C-NE are displayed in Figure 3. The I3C-loaded *DM* ZnO nanoemulsion showed physiognomies peaks of functional groups of *DM* extract, ZnO NPs, and I3C. The *DM*-ZnO-I3C-NE exhibited a broad peak at 3377.63 cm^−1^ representing the phenolic (–OH) and secondary amine (–NH) group due to the polyphenol from *Dendropanax* extract and secondary amine (–NH) of I3C [24,25]. Absorbance at 2850 cm^−1^ and 1383 cm^−1^ represents the functional group of medium (–CH) stretch and (–C=O) stretching. The *DM*-ZnO *NPs* and *DM* extract exhibit functional groups at 3382–3271 cm^−1^, also representing the phenolic (–OH) group from the polyphenol compound of the plant extract. The DM-ZnO NPs also demonstrated an absorption peak at 1598 cm^−1^ observed in plant extract, which may be due to the presence of (–OH), (–CH) groups. I3C showed a narrow peak at 3379 cm^−1^ due to the presence of the (–NH) secondary amine bond and also absorption bands at 2800–3000 cm^−1^ and 1450–1554 cm^−1^ representing C–H stretching and C=C stretching bond for alkyne molecules. Finally, the *DM*-ZnO NPs and *DM*-ZnO-I3C-NE both contain an IR transmission peak at (450–500) cm^−1^, indicating the presence of metal ZnO NPs and confirming the successful formation of *DM*-ZnO-I3C-NE from plant extract mediated with *DM*-ZnO NPs and I3C [26,27].

### 3.4. XRD Analysis

**Figure 4:**
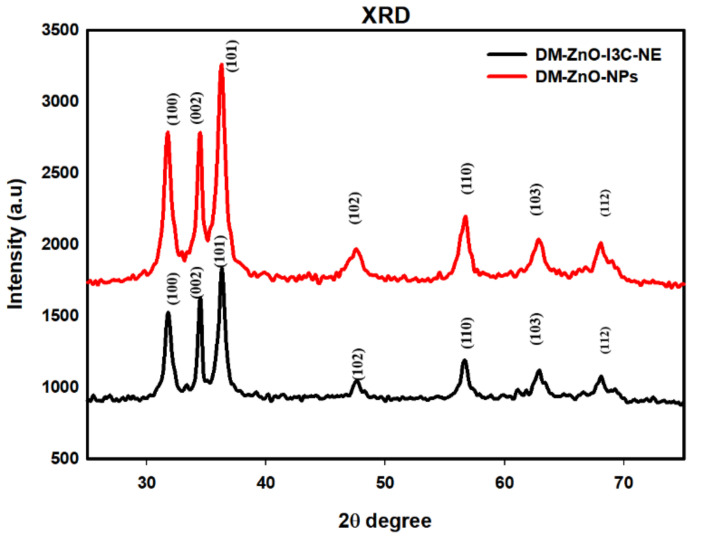
XRD analysis for *DM*-ZnO NPs (Red) and *DM*-ZnO-I3C-NE (Black).

The crystalline structure and crystallite size insight into the nanomaterial are provided through the XRD analyzer. The XRD pattern of the synthesized DM-ZnO NPs and DM-ZnO-I3C-NE is shown in Figure 4. The XRD peaks showed strong diffraction peaks at 31.810, 34.450, 36.290, 47.770, 56.670, 62.890, 67.990 and 31.780, 34.470, 36.290, 47.620, 56.690, 62.890, and 68.050 corresponds to the lattice crystal planes (100), (002), (101), (102), (110), (103), and (112), which had significant agreement with JCPSD card no. 89–1397 [28]. The most intense peak at 36.290 belongs to (101) orientation, and also, its position reflects the hexagonal wurtzite structure of both cases. The presence of (100), (002), and (101) indicated the high purity of the nanoformulation and also indicated that there are no other impurities. The strong and narrow peak indicated the highly crystal nature for both cases.

The average crystalline size of the nanoparticles and nanoemulsion were calculated using Scherrer Equation (1), which is also shown in Table 2 and Table 3.

(1) $$D=\frac{k\lambda }{\beta \cos0}$$

where *D* is denoted as the crystalline size of the particles, *k* is the Scherrer constant, which is equal to the shape factor 0.9, the wavelength of light diffraction is *λ* where (*λ* = 1.54 Å), *β* is the FWHM (full width at half maximum), and *θ* is the Bragg angle of refraction.

The crystalline sizes were calculated for both *DM*-ZnO NPs and *DM*-ZnO-I3C-NE, and the nanoemulsion is 11.52 nm and 16.07 nm, which are respectively shown in Table 2 and Table 3. The sharp and clear peaks established the highly purity and crystalline flora of the *DM*-ZnO NPs and *DM*-ZnO-I3C-NE [29]. The XRD pattern highly support the successful loading of ZnO NPs into the nanoemulsion.

### 3.5. FE-TEM, Elemental Mapping, and FE-SEM Analysis

**Figure 5:**
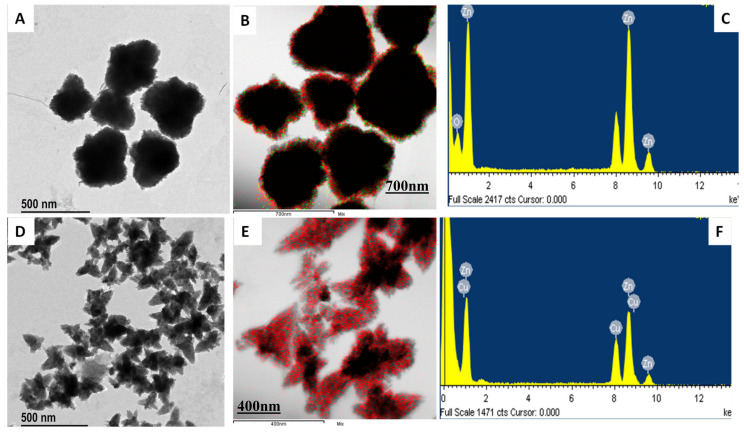
FE-TEM analysis of *DM*-ZnO nanoparticles, (**A**) Multiple images at 500 nm bar range (no aggregation), (**B**) Elemental mapping and *DM*-ZnO-I3C nanoemulsion, (**C**) EDS (Energy dispersive spectroscopy) of DM-ZnO nanoparticles, (**D**) Multiple images for DM-ZnO-I3C-NE at 500 nm range, (**E**) Elemental mapping, (**F**) EDS of *DM*-ZnO-I3C nanoemulsion.

The morphology of the nanoemulsion was characterized using FE-TEM. ZnO NPs showed the spheroid nanoparticle in a 500 nm scale range that did not aggregate with each other (Figure 5A). In contrast, in *DM*-ZnO NPs loaded with I3C using the oil-in-water system, the morphology changed to a flower shape with systematic orientation, and the entrapment of I3C was confirmed as successful with *DM*-ZnO in the 500 nm range (Figure 5D,E). Inclusion of the hydrophobic part helps it become stable and encircled by the hydrophilic part of the surfactant, which also enclosed the lipid layer of olive oil. In higher magnifications, I3C showed a nano-flower structure with 3–4 broad petal-like structures. The flower shape refers to the geometrical resemblances to a flower, with pyramid-like petal dimensions of less than 100 nm [30]. Flower-like nanostructures can disrupt the cell wall and cell mechanisms better than spherical shapes, thus promoting the death of human endothelial cells [31,32]. Therefore, the flower shape indicates that this substance can be a potential candidate for cancer drug delivery. The EDX (Energy-dispersive X-ray spectroscopy) mapping indicated the presence of Zn and O in the ZnO nanoformulation (Figure 5B,E). The peak signal of zinc and oxygen successfully confirmed the formation of ZnO nanoparticles in Figure 5C.

**Figure 6:**
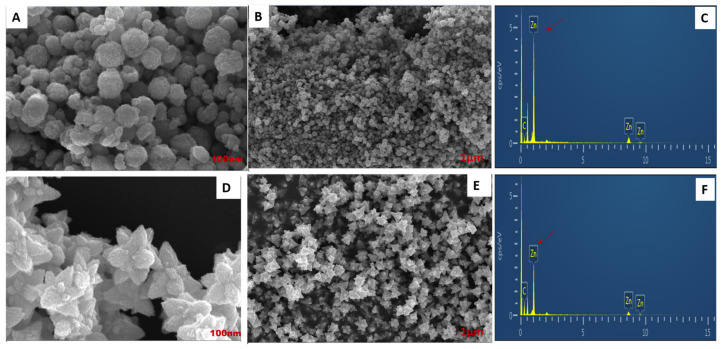
FE-SEM analysis of *DM*-ZnO nanoparticles exhibited a spheroid shape in (**A**), (**B**) and *DM*-ZnO-I3C nanoemulsion exhibited a flower shape in (**D**), (**E**) EDS analysis for *DM*-ZnO NPs (**C**) and *DM*-ZnO-I3C-NE (**F**).

FE-SEM morphology further confirmed that the *DM*-ZnO spheroid (Figure 6A,B) showed no aggregation and also that *DM*-ZnO-IC3-NE showed a flower-shaped morphology, which coincides well with the FE-TEM data (Figure 6D,E). The presence of Zn and O were identified in the EDS spectra through a sharp peak signal of both metals, indicating the formation of zinc oxide nanoparticles and nanoemulsion and also confirming no aggregation.

### 3.6. Size Measurement Analysis

**Figure 7:**
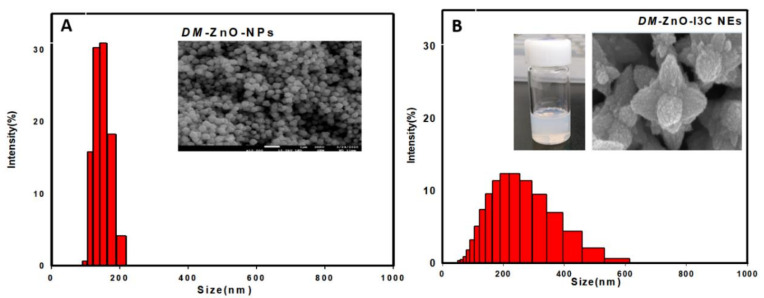
Size distribution analysis using dynamic light scattering (DLS) analyzer for (**A**) *DM*-ZnO NPs (**B**) *DM*-ZnO-I3C NE.

DLS analysis is essential for determining the hydrodynamic size of the nanoparticles and nanoemulsion. The hydrodynamic diameter and PDI value of *DM-ZnO NPs* is about 65 ± 3 and 0.50 nm, respectively (Figure 7A,B). When I3C was entrapped using the oil-in-water method, the obtained nanoemulsion increased in size to 239.6 ± 6.13 nm with a PDI value of 0.13 due to the entrapment of new hydrophobic compounds. The stability of the nanoemulsion was analyzed using a zeta analyzer; the *DM*-ZnO-I3C-NE zeta potential observed was around −12 ± 0.5 MeV, which confirmed the nanoemulsion stability. The small PDI value indicated the monodispersibility of both nano compositions, which was further confirmed by FE-TEM and FE-SEM.

### 3.7. In Vitro Cell Cytotoxicity Analysis

**Figure 8:**
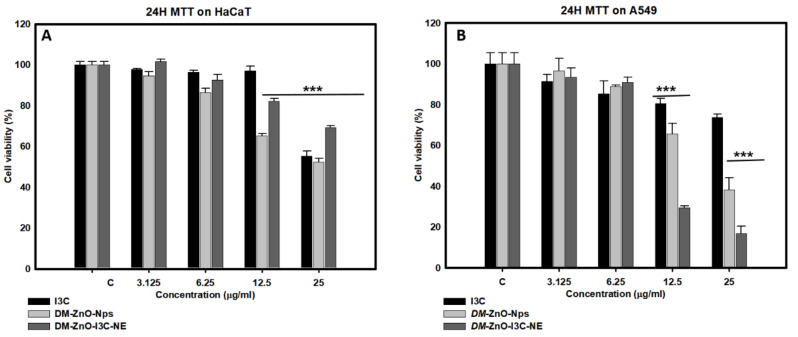
In vitro cytotoxicity analysis for I3C, *DM*-ZnO NPs, and *DM*-ZnO-I3C-NE nanoemulsion in (**A**) Human keratinocyte cells (HaCaT) and Human lung carcinoma (A549) cells (**B**). Each value is expressed as the mean ± standard error of three independent experiments. *** *p* < 0.001 compared with control.

Non-cancerous HaCaT cells and A549 lung cancer cells were treated with *DM*-ZnO-I3C nanoemulsions in different concentrations (0, 3.125, 6.25, 12.5, and 25 µg/mL) for 24 h. Cytotoxicity analysis was determined using MTT assay (Figure 8). We performed HaCaT as a control normal cells as per the previously reported papers [33]. Recent research found HaCaT cells were considered one of strongest for ACE2 receptors which is prone to be more susceptible for lung [34]. This encourages more investigation using these cell lines. The *DM*-ZnO-I3C nanoemulsion showed diverse effects on killing A549 cells and had no impact on HaCaT cells in a dose-dependent manner for up to 24 h. *DM*-ZnO-I3C-NE significantly induced cancer cell growth of more than 80% at a concentration of 12.5 µg/mL after 24 h treatment. In contrast, *DM*-ZnO-I3C-NE showed minimum toxicity on normal cells after 24 h treatment. This cytotoxicity result was similar to a previous study [35]. We performed similar assays using free *DM*-ZnO and I3C. *DM*-ZnO-I3C-NE showed significantly more toxicity in cancerous cells than the other two samples; this may be due to the synergistic effect of the nanoemulsion prepared using DM ZnO NPs and the ability to deliver I3C at the target site. The increase in the cell toxicity clearly shows that the drug I3C is effectively delivered at the target site and due to the formation of nanoemulsion made. Additionally, the presence of dissolved Zn ion helps for the mitochondrial and DNA damage due to the dissolved metal ions and allows the drug to effectively block the tumor growth. This is well supported by the MTT results where the DM-ZnO NPs is less toxic than the DM-ZnO-I3C NEs in A549 cell line. Similarly, the I3C toxicity at A549 suggests that the drug is not sufficiently reached to increase its capabilities. This suggests that the drug carrier using the plant extract with a metal ion and capping agent is an effective control for this synergetic effect.

### 3.8. ROS Generation Induced by DM-ZnO-I3C-NE

The anti-cancer activity of *DM*-mediated Ag and Au NPs were previously reported [36,37]. Nanosized ZnO was also shown to induce the toxicity of human lung carcinoma cells [38]. However, the anti-cancer activity *DM*-ZnO nanoparticles and *DM*-ZnO-I3C nanoemulsion on lung cancer cells have not been reported. In this study, *DM*-ZnO-I3C was synthesized and checked for anti-cancer activity against A549 human lung carcinoma cells. MTT assays revealed a high toxicity of the *DM*-ZnO-I3C nanoemulsion on A549 lung cancer cells at 12.5 µL/mL concentration. In contrast, *DM*-ZnO-I3C exhibited minimum toxicity in normal cell lines in the same conditions. Therefore, the determination of the anti-cancer effect of *DM*-ZnO-I3C has been done evaluating cytotoxicity together with ROS level measurement.

**Figure 9:**
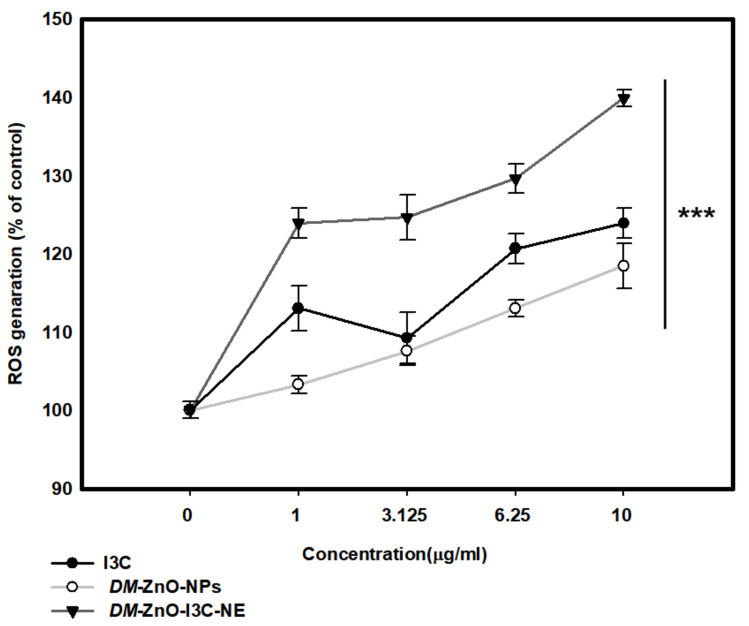
Reactive oxygen species (ROS) generation ability of *DM*-ZnO-NE with compared free drug in A549. Each value is expressed as the mean ± standard error of three independent experiments. *** *p* < 0.001 compared with control.

Studies have shown that nanomaterials induce cell death due to oxidative stress [39]. As shown in Figure 9, *DM*-ZnO-I3C produced a higher ROS level than free *DM*-ZnO and I3C alone at 10 µg/mL. It was also found that 10 µg/mL can change cellular morphology [40]. ROS generation due to ZnO nanoparticles was investigated in detail; however, no mechanism has been clearly elucidated.

The cell cytotoxicity is apparent due to the insolubility of ZnO nanoparticles to free Zn^2+^ due to its higher concentration. The potential therapeutic approach due to the transition metals can regulate the redox cycling cascades to generate the ROS levels due to the presence of H_2_O_2_. This phenomenon has been previously shown by incorporating the Fe^3+^ ions with zinc metal, which correlates the Fenton’s reagent mechanism of enhanced ROS levels in cancer cells [41]. Higher ROS generation may be due to the formation of nanoemulsion, while an active component is more protective from physiological stress than free drug and also shows an increased surface area, which will increase drug bioavailability [42]. These results suggest that the *DM*-ZnO-I3C nanoemulsion may be a new drug and a green source for lung cancer treatment.

### 3.9. Effect of DM-ZnO-I3C-NE on Cell Morphology

**Figure 10:**
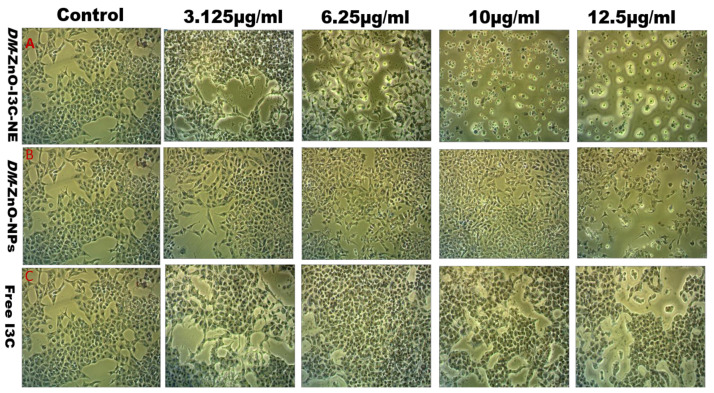
Optical microscopic image for morphology changes of A549 lung cancer cells after treated with *DM*-ZnO-NE 24 h compared with control. (**a**) *DM*-ZnO-I3C-NE; (**b**) *DM*-ZnO NPs; (**c**)Free I3C.

We investigated the influence of *DM*-ZnO-I3C-NE compared with free *DM*-ZnO *NPs* and I3C drugs on A549 lung cancer cell morphology under an optical microscope. We observed the morphological changes in A549 cells due to the excessive oxidative stress caused by *DM*-ZnO-I3C NE. Compared with untreated controls, the cell density of treated cells was drastically reduced, and the cell shape showed a different profile based on dose for time. Then, we investigated the morphology after 24 h. The morphological differences may be due to the shift from epithelial to mesenchymal expression change approaching the fibroblast-like expression [43]. Figure 10C shows the morphological changes with different concentrations of I3C drug, and Figure 10B shows results for *DM*-ZnO NPs, while Figure 10A shows the synergetic effect of Zn^2+^ ions and I3C drug of *DM*-ZnO-I3C NE. The results indicate that the cell morphology alterations may be due to ROS generation caused by Zn^2+^ ions and I3C and oxidative stress [44]. This result is well coincident with our ROS generation study. Interestingly, the components for this nanoformulation had a similar property at the target site, and thereby the synergistic effect was confirmed.

## 4. Conclusions

*DM* is an ancient traditional tree that is endemic in South Korea. Here, excitingly, a 100-year-old *Dendropanax* plant extract is used that had been demonstrated to show various therapeutic efficacies. Our study represents a facile approach for the biological synthesis of a functional nanoformulation using *DM* plant extract-mediated ZnO nanoparticles loaded with the anti-cancer drug I3C based on O/W nanoemulsion using an ultrasonication process. Thus, we designed an oil-in-water nanoemulsion where I3C was easily entrapped into the lipophilic part as well as *DM*-ZnO NPs with the highly enriched active component of *Dendropanax* producing an outer hydrophilic layer with the help of a non-ionic surfactant to enhance the efficacy of active ingredients. This outer capping ensures the safe carrier system for Zn^2+^ ion and I3C drug to the respective site. Upon exposure to the disease site, the outer core layer degrades slowly and allows the Zn metal to be exposed to the acidic environment, leading to the mitochondrial disruption due to transition permeability and thereby causes the oxidative stress, which leads to apoptosis. During this phenomenon, the bioactivity of I3C also induces and causes the synergetic effect to I3C drug for its ROS generation capabilities. This increase in the dual functionality enables a faster and effective way of blocking the tumor and enhancing the rapid activity at the tumor site. The increase in the ROS generation in the *DM*-ZnO-I3C-NE particles suggests the synergetic response of I3C and Zn2+ ion. In vitro cytotoxicity analysis revealed the anti-cancer efficacy of *DM*-ZnO-I3C-NE on a human lung cancer cell line (A549) as well as the production of ROS. *DM*-ZnO-I3C-NE induced higher ROS levels compared with free *DM*-ZnO and I3C on human lung carcinoma cells. The results of ROS generation validated with cell morphological studies as the higher concentration of the *DM*-ZnO-I3C-NE was found to be effective for apoptosis against the free I3C and *DM*-ZnO NP. These results suggest the efficacy of the compounds at the target site and open a door for detailed investigation of the compound mechanisms.

*DM*-ZnO-I3C-NE showed lower toxicity in the HaCaT human keratinocyte cell line. The increased ROS level and toxicity on human carcinoma lung cancer cells by *DM*-ZnO-I3C-NE indicates it may be a potential source of future nano-drugs for lung cancer treatment. This approach also provides a novel nanoformulation for functional food, which is a proven safe and efficient approach for commercial applications.

## Figures and Tables

**Table 1:** The optimization process for nanoemulsion preparation.

Sample (Name)	*DM*-ZnO NPs (Aqueous Solution)	Olive Oil	I3C Drug	Surfactant (Tween 80)
S1	82% (37.5 mg)	10%	5% (12.5 mg)	3%
S2	80% (37.5 mg)	8%	5% (12.5 mg)	7%
S3	80% (37.5 mg)	5%	5% (12.5 mg)	10%

**Table 2:** Crystalline size analysis of *DM*-ZnO-Nps using an XRD analyzer. FWHM: full width at half maximum.

Lattice Plane	Position of Peak (2θ)	d-Spacing Value (A^0^)	FWHM Value (2θ)	Size (nm)	Average (nm)
100	31.81	d = 2.81	0.8242	10.03	
002	34.45	d = 2.60	0.5691	14.63	11.52
101	36.29	d = 2.47	0.8438	9.92	
102	47.77	d = 1.90	2.3549	3.69	
110	56.67	d = 1.62	1.3541	6.67	
103	62.89	d = 1.48	1.9036	4.90	
112	67.99	d = 1.37	2.7474	3.49	

**Table 3:** Crystalline size analysis of *DM*-ZnO-I3C-NE using an XRD analyzer.

Lattice Plane	Position of Peak (2θ)	d-Spacing Value (A^0^)	FWHM Value (2θ)	Size (nm)	Average (nm)
100	31.78	d = 2.80	0.7261	11.39	
002	34.47	d = 2.59	0.3336	24.95	16.0708
101	36.29	d = 2.47	0.7065	11.84	
102	47.62	d = 1.90	2.5904	3.35	
110	56.69	d = 1.62	1.1382	7.94	
103	62.89	d = 1.47	2.0606	4.52	
112	68.05	d = 1.37	2.1587	4.44	
